# Redefining the role of the thiol-based agent *N*-acetylcysteine in human health and disease and elucidating potential advantages of its amide derivative

**DOI:** 10.1039/d5md01173f

**Published:** 2026-04-01

**Authors:** Hui-Qi Qu, Charlly Kao, Hakon Hakonarson

**Affiliations:** a The Center for Applied Genomics, Children's Hospital of Philadelphia 3615 Civic Center Blvd, Abramson Building Philadelphia Pennsylvania 19104 USA hakonarson@chop.edu +1 267 426 0363 +1 267 426 0088; b Department of Pediatrics, The Perelman School of Medicine, University of Pennsylvania Philadelphia Pennsylvania 19104 USA; c Division of Human Genetics, Children's Hospital of Philadelphia Philadelphia Pennsylvania 19104 USA; d Division of Pulmonary Medicine, Children's Hospital of Philadelphia Philadelphia Pennsylvania 19104 USA; e Faculty of Medicine, University of Iceland 101 Reykjavik Iceland

## Abstract

*N*-Acetylcysteine (NAC) is the established antidote for acetaminophen toxicity and an approved mucolytic agent. Beyond these traditional uses, increasing evidence highlights its broader role as a modulator of thiol–redox biology. Rather than functioning as a nonspecific antioxidant, NAC modulates glutathione metabolism, redox-sensitive signaling, immune checkpoints, thiol-based post-translational modifications, ferroptosis susceptibility, and glutamatergic neurotransmission. This review synthesizes mechanistic, preclinical, and clinical evidence across pulmonary, hepatic, neuropsychiatric, metabolic, cardiovascular, and oncologic disorders, emphasizing how variability in baseline redox state, pharmacogenetics, and delivery contributes to heterogeneous outcomes. Strategies to improve pharmacokinetics and tissue targeting include structural derivatives such as *N*-acetylcysteine amide (NACA), and combination regimens such as NAC with probenecid or GlyNAC. Emerging applications span long COVID, neurodegeneration, psychiatric disorders, microbiome–redox interactions, environmental toxicology, and cancer immunotherapy. NAC and NACA exemplify the evolution of redox-targeted therapeutics. NAC is well established for safety and clinical utility, but its pharmacokinetic and tissue distribution properties constrain broader efficacy. NACA, a lipophilic amide derivative, enhances membrane permeability and cellular uptake, suggesting it may achieve higher tissue exposure at lower doses. Future progress will rely on biomarker-guided, precision approaches that optimize dosing, formulation, and delivery while exploring rational combinations across disease contexts defined by redox biology.

## Abstract 2

*N*-Acetylcysteine amide (NACA) reduces polarity and ionization relative to NAC, improving membrane permeability and intracellular exposure while preserving thiol-mediated redox activity and glutathione support.

## Graphical abstract

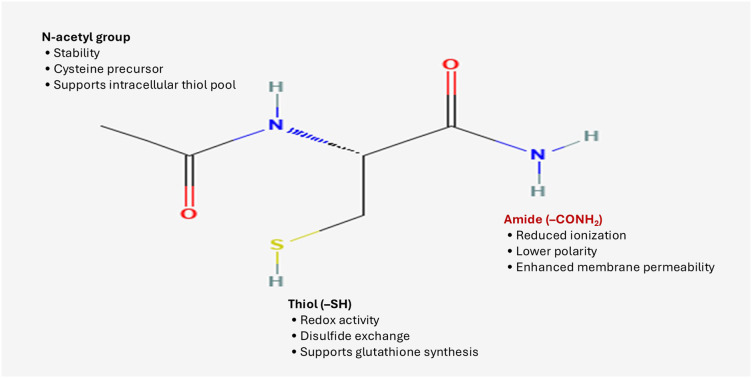

## 1. Introduction

*N*-Acetylcysteine (NAC) straddles two distinct roles: as an established therapeutic agent and as a dietary supplement. Clinically, it is the standard antidote for acetaminophen (APAP) overdose, restoring hepatic glutathione (GSH) and preventing or mitigating drug-induced liver injury and lowering risk of acute liver failure when administered within the ideal time window.^1,2^ It is also approved as a mucolytic in respiratory disease. Off-label, NAC has been explored in a variety of oxidative stress-related conditions, among them psychiatric, hepatic, metabolic, and pulmonary.^1^

Yet the traditional framing of NAC, as merely a nutrient, glutathione precursor, or general antioxidant, fails to capture its full potential. NAC's thiol group enables it not only to act directly as a reducing agent but also to influence upstream regulation of redox balance, signaling, immune function, and cell fate decisions.^3^ For example, in APAP hepatotoxicity, NAC does more than simply replenish GSH, it supports mitochondrial recovery, reduces oxidative/nitrosative damage, and influences metabolic fluxes.^4^

A relevant parallel is the arginine–NO axis: arginine is both a basic amino acid essential for protein synthesis and, through nitric oxide synthase, the substrate for regulated NO production with far-reaching vascular, immune, and metabolic effects.^5^ Analogously, NAC sits upstream of the cysteine–GSH axis, affecting cystine/cysteine transport [*e.g. via* SLC7A11, the cystine–glutamate antiporter (xCT)], influencing GSH biosynthesis, and thereby altering redox proteome states and downstream redox-sensitive signaling.^6,7^

**Fig. 1:**
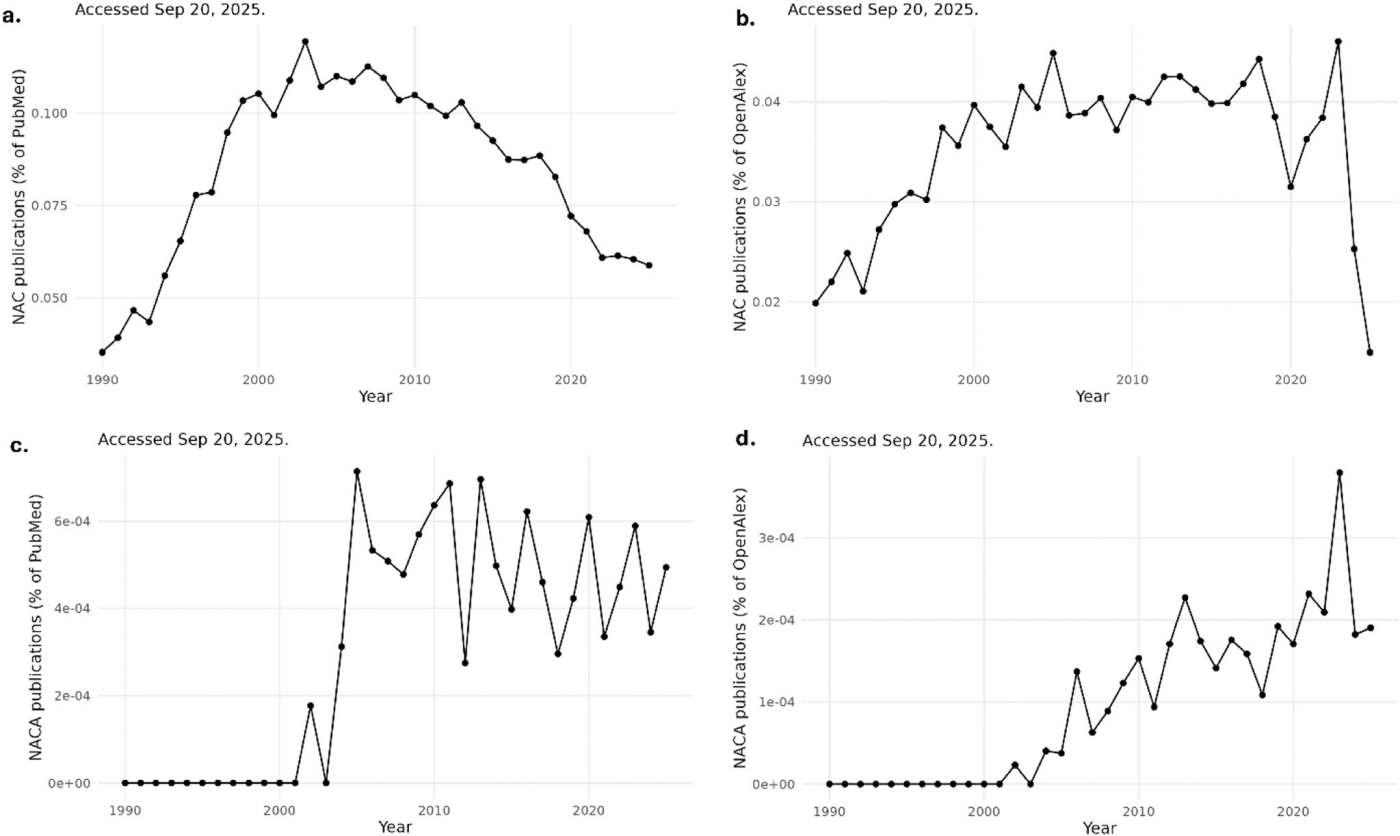
Trends in NAC/NACA research and clinical testing. (a) Annual NAC-related publications as a percentage of all records indexed in PubMed, 1990–2025. (b) Annual NAC-related publications as a percentage of all records indexed in OpenAlex, 1990–2025.^91^ (c and d) Annual NACA-related publications as a percentage of all PubMed records (c) and OpenAlex works (d), 1990–2025. All searches were performed on September 20, 2025, using harmonized query terms and de-duplication procedures. Data were retrieved *via* easyPubMed^92^ and openalexR,^93^ then parsed using dplyr/tidyr^94^ and visualized with ggplot2.^95^

Research momentum also reflects this broadened view. Bibliometric surveys consistently show that NAC has transitioned from a niche research topic in the 1980s and 1990s to a high-volume field today. Global publication output has risen substantially over the past three decades, with annual records increasing several-fold between the early 2000s and the 2020s (Fig. 1a and b). Domain-specific analyses echo this expansion: focused bibliometric studies have identified sustained growth of NAC publications in neurology, psychiatry, oncology, and respiratory medicine, with particular acceleration in neurodegeneration and neuroprotection.^8,9^ ClinicalTrials.gov mirrors this trend. Using the ClinicalTrials.gov API v2, we identified 1101 registered trials mentioning NAC, out of a total of 553 942 clinical trials, corresponding to about 0.2% of all trials in the registry. These bibliometric signals and trial-registration counts reflect broad and expanding investigative interest in NAC across mechanistic and clinical contexts, including studies that explicitly interrogate thiol–redox pathways.^8,9^ However, publication and trial counts primarily reflect the level of research attention devoted to a topic and should therefore be interpreted cautiously. They provide limited insight into study quality, endpoint rigor, or reproducibility. In this review, these indicators are used only to illustrate the scale and diversification of NAC-related research activity, whereas assessment of therapeutic relevance rests primarily on mechanistic evidence and clinical studies.

In this review, we examine NAC as a modulator of thiol–redox biology and consider the translational factors that shape when thiol-based interventions succeed or fail. We outline the chemical and pharmacological foundations of NAC, including its nutritional context and human pharmacokinetics, with attention to determinants of systemic exposure and interindividual variability. Structural derivatives and combination strategies designed to improve pharmacokinetics and tissue targeting are also considered, particularly *N*-acetylcysteine amide (NACA) and selected combination approaches. Mechanistic sections synthesize pathways central to thiol–redox regulation, including glutathione metabolism and mitochondrial function, redox-sensitive signaling networks, immune redox checkpoints, ferroptosis susceptibility, thiol-based post-translational modifications, and xCT-linked glutamatergic modulation. Clinical evidence is evaluated across major disease domains, including pulmonary and hepatic disorders, neuropsychiatric and neurodegenerative conditions, cardiometabolic disease, and oncology, with emphasis on sources of heterogeneity and opportunities for biomarker-guided stratification. Preclinical studies suggest that NACA, a more lipophilic amide derivative, may have improved membrane permeability and tissue penetration compared with NAC.^10,11^ Across indications, many studies are limited by small samples, heterogeneity of endpoints, reliance on surrogate biomarkers, and nonrandomized designs.^12^ Throughout this review we separate mechanistic plausibility from clinical validation, and aim at grading conclusions, accordingly.

## 2. Nutrition and pharmacology

NAC is the *N*-acetylated derivative of cysteine. Its free thiol (–SH) group drives disulfide exchange with protein cysteines and reductive reactions that underpin both its mucolytic activity and its redox effects in cells. After absorption, NAC can be deacetylated to cysteine, thereby supplying substrate for GSH synthesis and the wider cellular thiol pool.^3^ Clinically, this thiol reactivity also explains its mucolytic effect, which arises from the reduction of disulfide bonds within mucin glycoproteins, decreasing mucus viscosity and improving clearance.

From a nutritional standpoint, NAC is not a natural food constituent; rather, cysteine and its precursor methionine are obtained from dietary protein (meat, eggs, dairy, legumes, nuts/seeds) and sulfur-rich vegetables (alliums, crucifers). Human data indicate that habitual intake of cysteine tracks with endogenous antioxidant capacity: in a cross-sectional study (*n* = 41) higher dietary cysteine strongly correlated with erythrocyte GSH (*r* = 0.765, *p* < 0.001) and lower F2-isoprostanes.^13^ In older adults, greater protein intake increased erythrocyte GSH synthesis with evidence for a threshold beyond which additional protein yielded diminishing returns.^14^ Nutritional interventions can shift tissue GSH: a randomized controlled trial in low-dairy consumers (aged 60–89) showed that increasing milk to ∼3 cups per day for 12 weeks raised brain GSH concentrations measured by magnetic resonance spectroscopy (MRS).^15^ Cysteine-rich protein sources can also augment systemic GSH in deficiency states. For example, whey protein supplementation increased plasma GSH in patients with advanced HIV infection.^16^ However, dietary cysteine supply depends on protein digestion, intestinal absorption, and transport, and is constrained by metabolic bottlenecks such as first-pass metabolism in the gut and liver and competition among amino acid transporters.^17,18^ Cysteine is considered a semi-essential amino acid because requirements increase under conditions of oxidative stress, inflammation, or rapid growth, where endogenous synthesis and dietary intake may be insufficient.^18^ Compared with dietary cysteine, which depends on digestion, intestinal absorption, and competition among amino acid transporters, NAC supplementation bypasses these upstream steps by being deacetylated to cysteine after uptake. Although its oral bioavailability is limited (∼6–10%) by first-pass metabolism, NAC still provides a more direct and pharmacological means of augmenting the intracellular cysteine pool when physiological demand exceeds nutritional capacity.^3^

Pharmacokinetically, the essential features of NAC have been known for decades: after oral dosing, peak plasma concentrations occur within 1–2 hours, but presystemic metabolism in the gut and liver leads to low systemic bioavailability, while intravenous dosing achieves immediate therapeutic levels.^19^ Clinically, these pharmacological properties underpin its two best-established applications: as a mucolytic when delivered by inhalation and as the standard intravenous antidote for acetaminophen poisoning, administered *via* the widely adopted three-bag regimen. These classic insights frame NAC as a compound with limited oral bioavailability and route-dependent systemic exposure and clinical utility.^3,19^ More recent human studies have refined this picture. In a randomized cross-over trial in 30 healthy Chinese and Caucasian volunteers, a single 600 mg oral dose of NAC reached peak plasma concentrations within approximately 1 h, and repeated twice-daily dosing for 3 days led to modest accumulation (ratio 1.4–1.5). The elimination half-life after repeated dosing was substantially longer than historically reported, averaging 15.4 h in Chinese participants and 18.7 h in Caucasians, and only ∼3–4% of the dose was excreted unchanged in urine, underscoring the extent of metabolism.^20^ In critically ill patients with pneumonia, sepsis, or isolated brain injury, enteral NAC displayed delayed absorption and altered clearance compared with healthy subjects, highlighting the impact of disease states on systemic exposure and variability.^21^

Renal function may also contribute to pharmacokinetic heterogeneity. Although only a small fraction of NAC is excreted unchanged in urine,^20^ kidney function still influences NAC pharmacokinetics. Evidence indicates that total NAC clearance is reduced in advanced chronic kidney disease (CKD), resulting in altered systemic exposure compared with individuals with preserved renal function.^22^ CKD is also characterized by disturbances in redox homeostasis, including glutathione deficiency and altered Nrf2-dependent signaling, which may modify cellular responses to thiol-based therapies such as NAC.^22^ These pathophysiological features reinforce the importance of considering renal function when extrapolating dose–exposure–response relationships across disease states. Collectively, variability in NAC response reflects not only dosing regimen and route of administration but also underlying physiology, including kidney function.

These data emphasize that, although the foundational pharmacology of NAC is well established, oral bioavailability and clearance vary significantly with dosing regimen, ethnicity, and disease state. Such variability underscores the importance of pharmacokinetic stratification in clinical trial design and precision medicine applications.

Pharmacodynamically, NAC replenishes cysteine to drive GSH biosynthesis when cysteine is rate-limiting, restoring the GSH/GSSG ratio and improving cellular redox buffering. Nutritional studies illustrate that diet alone can modulate GSH in blood and brain, while pharmacological NAC supplies supraphysiologic cysteine equivalents that can overcome deficits induced by aging, malnutrition, or oxidative stress.^13,15^ The deficits are well documented: aging is associated with declining GSH levels and impaired cysteine availability in multiple tissues, contributing to increased vulnerability to oxidative damage.^23^ Malnutrition, especially protein-energy deficiency, limits methionine and cysteine intake, leading to suppressed GSH synthesis and weakened redox buffering.^24^ Oxidative stress, whether from chronic disease, environmental exposures, or infection, further depletes cysteine pools and drives GSH consumption.^25^ In these contexts, dietary supply may be insufficient to restore redox balance, whereas NAC bypasses metabolic bottlenecks and provides an immediately available cysteine donor to replete intracellular thiol stores.

Dose–response and tolerability vary by route and context. Orally, NAC is generally well tolerated, with gastrointestinal complaints the most common adverse effects, and more common at higher doses. Intravenous administration can provoke non-IgE anaphylactoid reactions (flushing, pruritus, nausea, bronchospasm), whose incidence and risk factors have been quantified in large cohorts; reactions are more frequent at lower presenting acetaminophen concentrations and with faster infusions, and can usually be managed without discontinuation.^26^ Collectively, these data position nutrition as the physiological substrate for thiol/redox homeostasis, with NAC functioning pharmacologically to surpass dietary constraints, restore GSH and thiol pools, and buffer oxidative stress when higher-order control of the cysteine–GSH axis is therapeutically advantageous.

Following administration, NAC is readily absorbed but undergoes extensive first-pass metabolism in the liver and gut. Its principal metabolic step is enzymatic deacetylation, mainly by acylase I and related aminoacylases, which converts NAC to cysteine. In parallel, NAC can form cystine and mixed disulfides (*e.g.*, NAC–cysteine, NAC–GSH adducts), which are transported into cells and reduced back to cysteine.^3^ Thus, NAC serves both as a direct thiol donor and as a reservoir that replenishes intracellular cysteine for downstream redox and signaling pathways.

## 3. NAC derivatives, combination compounds, and related thiol agents

**Fig. 2:**
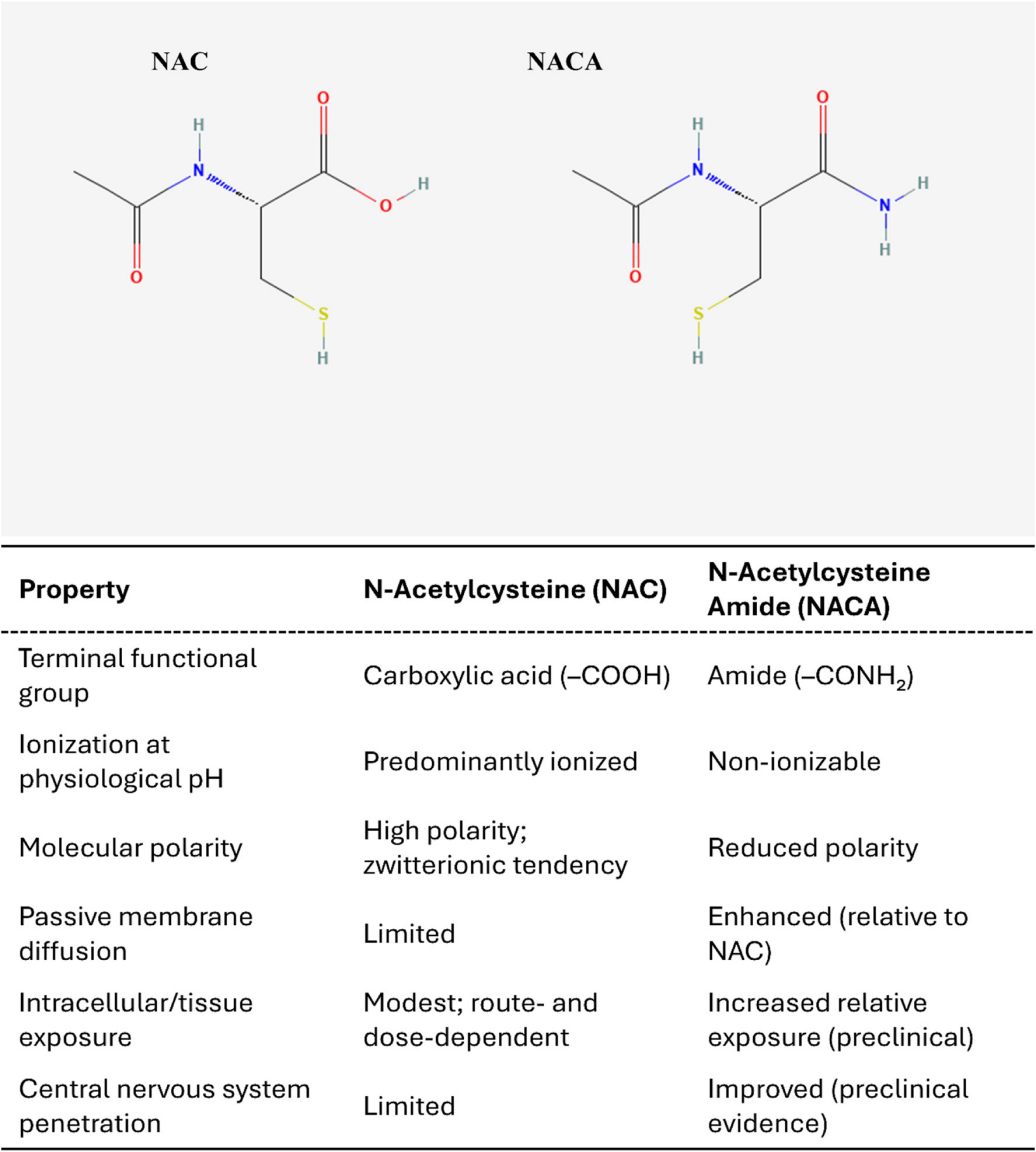
Chemical structures of NAC and NACA. NAC (PubChem CID 12035) contains a terminal carboxylic acid group (–COOH), while NACA (PubChem CID 10176265) is its amide derivative, with the –COOH replaced by an amide group (–CONH_2_). This single functional-group modification reduces ionization and polarity, increasing lipophilicity and enhancing passive membrane permeability relative to NAC, with preclinical evidence suggesting improved tissue and central nervous system access. Chemical structures were obtained from PubChem (National Center for Biotechnology Information, National Library of Medicine, National Institutes of Health).

NAC has important pharmacological limitations. Oral absorption is constrained by first-pass metabolism, its plasma half-life is short, and tissue penetration, particularly into the brain, is limited.^3,27,28^ Distribution across organs is nonspecific, which can dilute efficacy, and high-dose administration may cause gastrointestinal intolerance or infusion-related reactions.^29^ These constraints have motivated the development of structurally related thiol agents and combination strategies designed to improve stability, bioavailability, tissue targeting, and mechanistic specificity. Among these, a promising derivative is NACA (Fig. 2), designed to improve upon NAC's pharmacokinetics by increasing lipophilicity and membrane permeability. NACA-related publications began to appear in the early 2000s, with PubMed showing modest but sustained annual activity since then, and OpenAlex capturing a clearer upward trajectory with more works indexed into the 2020s (Fig. 1c and d).

In several *in vitro* heavy-metal or oxidative stress models, NACA has shown greater protection than NAC (*e.g.*, in lead toxicity and glutamate-induced cytotoxicity) in terms of restoring intracellular GSH, reducing ROS, and reducing lipid peroxidation.^10,11^ In rat blast injury models, systemic NACA reduced blast-induced intracranial pressure^30^ and preserved blood–brain barrier integrity,^31^ providing proof of concept for its translational application in traumatic brain injury and neurodegenerative conditions. Although NACA largely remains confined to preclinical and early translational stages, there are two recent registered trials ongoing in cerebral amyloid angiopathy (EU CT#: 2023-503969-36-01, https://euclinicaltrials.eu/ctis-public/view/2023-503969-36-01) and Alzheimer's disease (EU CT#: 2024-519497-39-00, https://euclinicaltrials.eu/ctis-public/view/2024-519497-39-00), respectively. NACA's increased lipophilicity and cellular membrane permeability, together with preclinical evidence of neurovascular protection, support its prioritization for CNS-focused indications.^10,11,30,31^

Bucillamine, a cysteine derivative carrying two thiol groups, also exhibits stronger reducing capacity in biochemical assays than NAC and has been employed clinically in Japan and South Korea for rheumatoid arthritis for more than three decades.^32,33^ Its pharmacological activity derives from its dual thiol-donor properties. Preclinical studies suggest that, in certain oxidative stress models such as ischemia–reperfusion injury, bucillamine restores intracellular cysteine and GSH more effectively than NAC.^34^ Beyond rheumatology, bucillamine has also been discussed for potential repurposing because of its redox-modulating properties,^35^ though evidence outside autoimmune disease is limited to speculation and isolated case reports, without controlled clinical validation. In addition, its use is not without risk: case reports have described bucillamine-induced interstitial pneumonitis in susceptible patients,^36,37^ underscoring the importance of careful clinical monitoring for pulmonary toxicity.

Combination strategies have also been explored to augment NAC efficacy. Co-administration of NAC with probenecid, an inhibitor of organic anion transporters, has been shown in preclinical rat models to increase both systemic and brain exposure of NAC, and in a phase I clinical trial in children with severe traumatic brain injury to result in measurable NAC levels in cerebrospinal fluid when given together.^38^ In aging research, GlyNAC (glycine plus NAC) supplementation corrected GSH deficiency, improved mitochondrial function, and lowered oxidative stress in older adults over 24 weeks,^23^ providing evidence that paired thiol strategies may be more effective than NAC alone in targeting age-related redox imbalance. These derivatives and combinations highlight ongoing efforts to overcome NAC's pharmacological limitations and extend its therapeutic reach.

## 4. Mechanistic pathways of action

### 4.1 GSH replenishment and mitochondrial protection

Clinical and translational studies show that pharmacological administration of NAC or related cysteine precursors can correct GSH deficiency in vulnerable populations. In older adults, GlyNAC supplementation restored intracellular GSH levels, reduced oxidative stress, and improved mitochondrial function and systemic markers of health.^23^ In humans with schizophrenia or schizoaffective disorder, an 8-week randomized, placebo-controlled trial (2400 mg per day NAC) demonstrated increased medial prefrontal cortical GSH by MRS, with parallel trends on cortical glutamate, direct evidence that pharmacologic cysteine supply can raise brain antioxidant capacity *in vivo*.^39^ Complementing this, cell-based metabolic flux work shows NAC rescues GSH depletion and attenuates ROS-driven injury under glucolipotoxic stress, consistent with its role as a cysteine donor to GSH synthesis.^40^ Recent rodent studies further strengthen this: in cardiorenal syndrome type 4, NAC treatment (600 mg kg^−1^ per day) preserved mitochondrial bioenergetics, increased cardiac mitochondrial GSH, improved NAD^+^ levels, reduced H_2_O_2_ production, and elevated SIRT3 and SOD-2 activity in heart tissue.^41^ NACA reproduces these effects with enhanced intracellular delivery, restoring GSH and limiting mitochondrial oxidative damage at lower concentrations than NAC in neuronal and toxin-exposure models.^10,11^

### 4.2 Direct ROS/RNS scavenging

The free thiol (–SH) group of NAC confers intrinsic reducing activity, allowing it to directly scavenge certain reactive oxygen and nitrogen species, including hydroxyl radicals, hypochlorous acid, and peroxynitrite.^42,43^ Compared with enzymatic systems such as superoxide dismutase, catalase, and glutathione peroxidase, these reactions are kinetically less efficient and generally require millimolar NAC concentrations, which may occur locally in the airway lumen during mucolytic therapy or transiently after high-dose intravenous administration. Nonetheless, direct scavenging remains clinically relevant in these settings, complementing NAC's more durable effects mediated through cysteine supply and glutathione metabolism. In contexts where millimolar NAC is required for direct scavenging, NACA's improved cell entry can raise local thiol availability without escalating systemic doses.

### 4.3 Redox-sensitive signaling pathways (NF-κB, Nrf2, MAPKs)

Beyond bulk antioxidant defense, NAC influences redox-sensitive signaling pathways, which link oxidative stress to inflammation, antioxidant defense, and cell survival or death decisions. In primary human myeloid and airway cells, NAC blunted inflammasome activation and downstream cytokine release, aligning with suppression of ROS-dependent NF-κB signaling, a central regulator of inflammation and immune activation.^44^ While MAPK and Nrf2 pathways are classically redox responsive, contemporary oncology data in human primary cells and tumor models underscore how cyst(e)ine flux and xCT activity shape Nrf2 engagement: mesenchymal-like triple-negative breast cancer cells drive cystine uptake to activate NRF2 and sustain oxidative stress defenses, placing the cystine–GSH axis squarely at the interface of redox signaling and metabolic adaptation.^45^ By more efficiently repleting cysteine/GSH pools, NACA may modulate Nrf2, NF-κB, and MAPK nodes at lower exposures than NAC, although this remains to be established in human PK/PD studies.^10,11^

### 4.4 Immune redox checkpoints and T-cell metabolism

NAC engages immune metabolism by buffering ROS thresholds that gate T-cell activation and effector function. In translational models, NAC limited activation-induced T-cell death and improved antitumor T-cell efficacy, a principle now extended by studies demonstrating that cysteine/thiol support can enhance responsiveness to checkpoint therapy; in colorectal cancer models, acetylcysteine synergized with PD-1 blockade to potentiate antitumor immunity.^46^ In human inflammatory settings, NAC attenuated inflammasome-driven cytokine production and showed signals of dampening hyperinflammatory responses during COVID-19 care, consistent with modulation of cytokine storm biology.^44,47^ Given similar thiol pharmacology with better penetration, NACA may provide a more reliable lever on T-cell redox thresholds in tissues where NAC exposure is limited.

### 4.5 Ferroptosis regulation *via* the SLC7A11–GSH–GPX4 axis

Ferroptosis is a distinct, regulated form of cell death driven by iron-dependent lipid peroxidation. Unlike apoptosis or necrosis, it results from the uncontrolled accumulation of oxidized polyunsaturated fatty acids in cellular membranes, leading to catastrophic membrane damage and cell death.^48^ NAC intersects this process through the SLC7A11–GSH–GPX4 axis, a central protective pathway that controls cystine uptake, glutathione synthesis, and lipid peroxide detoxification by GPX4.^49^ Recent mechanistic work shows NAC rapidly replenishes intracellular cysteine and averts ferroptotic death, with evidence that GPX4 can leverage alternative reducing inputs under NAC rescue, clarifying why NAC can be potently anti-ferroptotic in mammalian cells.^49^ In disease models, inactivation of SLC7A11/GPX4 is sufficient to trigger ferroptosis and tissue injury, highlighting the axis that NAC modulates as a determinant of pathology and therapeutic response.^50^ Likewise, in neonatal intestinal injury (necrotizing enterocolitis), NAC was shown to reduce ferroptosis *via* downregulation of SESN2, improve epithelial barrier health, and suppress inflammation.^51^ These data sharpen the double-edged nature of NAC in oncology and neurodegeneration: cysteine repletion preserves vulnerable normal tissues but may also shield tumor cells from ferroptotic stress in biomarker-defined contexts. NACA should engage the SLC7A11–GSH–GPX4 axis comparably to NAC but with superior cellular access, a consideration for ferroptosis-related indications and for oncology contexts where anti-ferroptotic protection may be double-edged.

### 4.6 Thiol-based post-translational modifications (PTMs)

At the proteome level, thiol redox state governs reversible cysteine-centered PTMs such as *S*-glutathionylation, *S*-nitrosation, and sulfenylation that tune enzyme activity and signaling fidelity.^52^ Redox-proteomic advances now resolve proteome-wide thiol switches and their reversal by reducing inputs; recent data-independent acquisition mass spectrometry (DIA-MS) workflows captured broad remodeling of oxidized cysteines that was reversible with NAC, linking thiol PTMs to translational control and stress adaptation.^53^ In parallel, original studies in regenerative biology and neuroinflammation implicate stimulus-dependent *S*-nitrosation of nuclear^54^ and cytosolic targets^55^ as a functional signaling layer, establishing the PTM substrate that NAC, by shifting thiol redox, can indirectly modulate. Enhanced intracellular thiol buffering with NACA could more effectively reverse oxidized cysteine PTMs in targets that are poorly accessed by NAC.

Human data also support clinical relevance of thiol PTM modulation by NAC, particularly in the extracellular compartment. Human serum albumin Cys34 is the dominant circulating thiol and undergoes redox-dependent *S*-thiolation (*e.g.*, cysteinylation) under oxidative stress. In human plasma, NAC regenerates albumin Cys34 through thiol–disulfide exchange, shifting the balance from thiolated albumin toward native mercaptoalbumin, providing direct *in vivo* evidence that NAC can remodel a defined thiol PTM in humans.^56^ These findings support albumin Cys34 redox proteoforms as practical biomarkers of NAC target engagement and systemic thiol–redox effects. By contrast, systematic characterization of intracellular PTM remodeling in human tissues after NAC remains comparatively limited, underscoring the need for clinical redox-proteomic studies aligned with disease context and pharmacokinetic exposure.

### 4.7 Glutamatergic neurotransmission *via* xCT

NAC's neuromodulatory effects reflect its influence on xCT and glutamatergic homeostasis. In humans with cocaine dependence, a randomized cross-over ^1^H-MRS study showed that NAC reduced elevated anterior cingulate glutamate toward control levels, directly demonstrating modulation of glutamatergic tone *in vivo*.^57^ Mechanistically, preclinical work indicates that repeated NAC can normalize synaptic glutamate homeostasis *via* effects on GLT-1 and cystine–glutamate exchange, findings aligned with reduced cue-induced reinstatement in addiction models.^58,59^

Complementing these findings, more recent translational evidence shows that in substance-dependent individuals, NAC lowers elevated anterior cingulate glutamate/glutamine (Glx) and reduces cocaine-seeking behavior, consistent with normalization of synaptic glutamate cycling.^60^ In psychotic disorders, the trial that demonstrated cortical GSH repletion also showed shifts in glutamatergic tone, suggesting a mechanistic bridge between thiol metabolism and excitatory neurotransmission *in vivo*.^39^

Together, evidence from preclinical and clinical studies positions NAC as a systems-level modulator of the cysteine–GSH network: it replenishes GSH and mitochondrial defenses, directly scavenges ROS/RNS, reprograms redox-sensitive signaling (NF-κB/Nrf2/MAPKs), shifts immune redox checkpoints, modulates ferroptosis susceptibility *via* the SLC7A11–GSH–GPX4 axis, rewires thiol PTMs across the proteome, and tunes glutamatergic neurotransmission through xCT, with each effect contingent on baseline redox state, dose, route, and disease context. Because xCT-mediated cystine–glutamate exchange is compartment-specific, NACA's CNS penetration may amplify NAC-like normalization of glutamatergic tone in cortical and limbic circuits relevant to addiction and psychosis.

## 5. Therapeutic applications and precision health

Clinically, NAC has been established or explored across a wide spectrum of disorders, with applications spanning pulmonary, hepatic, neuropsychiatric, metabolic, cardiovascular, and oncologic diseases (Table 1).

**Table 1:** Mechanistic bases for NAC's therapeutic applications

Disease area	Core mechanistic actions of NAC	NACA-specific properties
Pulmonary diseases (COPD, cystic fibrosis, bronchiectasis, ALI, long-COVID)	Cleaves disulfide bonds in mucins (mucolysis); restores intracellular glutathione; suppresses airway oxidative stress and proinflammatory mediators (*e.g.*, IL-8, eosinophil cationic protein) *via* NF-κB inhibition; enhances antioxidant enzymes (*e.g.*, GPx) in bronchial and immune cells; rebalances mitochondrial bioenergetics and immune resilience in post-viral syndromes	Greater lipophilicity → improved tissue penetration and mitochondrial thiol delivery at lower doses
Hepatic medicine (APAP overdose, NAFLD/NASH)	Replenishes hepatic glutathione after acetaminophen overdose; supports mitochondrial recovery and hepatic blood flow; reduces nitrosative stress; activates the PGC-1α/SIRT1 axis to improve lipid metabolism; lowers fibrogenic mediators (*e.g.*, TIMP-1, PIIINP); pharmacogenetic interactions with GSH-pathway variants (*e.g.*, GCLC, GSTs)	Potential for improved oral exposure and hepatic delivery; clinical PK and tolerability remain to be defined
Neurology & psychiatry (addiction, schizophrenia, bipolar disorder, neurodegeneration, HCCAA)	Modulates cystine–glutamate exchange *via* xCT; normalizes cortical glutamate tone, reducing excitotoxic stress; raises cortical glutathione; suppresses microglial activation and NF-κB signaling, linking redox balance to neurotransmission; reduces ferroptotic death in stroke/TBI by sustaining GPX4 activity; disrupts cystatin C amyloid complex formation *via* direct thiol interaction, supporting targeted therapy in HCCAA	Preclinical evidence of enhanced CNS access and BBB-stabilizing effects; may improve CNS thiol repletion at lower concentrations
Endocrine & metabolic health (diabetes, obesity, insulin resistance)	Restores glutathione pools under metabolic stress; activates mitochondrial biogenesis through PGC-1α/SIRT1; reduces ROS-driven lipid peroxidation and inflammatory cytokines; improves insulin signaling and metabolic resilience; candidate biomarkers include HOMA-IR, cysteine/cystine redox balance, and oxidative stress indices (MDA, 4-HNE)	Better cellular uptake → improved correction of oxidative/mitochondrial stress in muscle and adipose tissue
Cardiovascular disease (endothelial dysfunction, atherosclerosis, hypertension risk)	Enhances nitric oxide bioavailability by reducing ROS; improves endothelial function and vascular redox balance; counteracts smoking-induced endothelial dysfunction; protects against LDL oxidation; modulates thiol–disulfide redox state of vascular proteins	Superior endothelial cell penetration; potentially greater impact on vascular redox balance
Oncology (chemoprotection *vs.* tumor support, immunotherapy synergy)	Protects normal tissues from chemotherapy-induced ROS and ferroptosis *via* cysteine supply and GPX4 activation; may inadvertently enhance tumor redox defenses in SLC7A11/NRF2-high cancers, contributing to chemotherapy resistance; modulates tumor immune microenvironment and synergizes with PD-1 blockade in preclinical models	Higher potency and tissue access; requires biomarker gating to avoid enhancing tumor redox defenses

### 5.1 Pulmonary diseases: COPD, cystic fibrosis, bronchiectasis, acute lung injury, and long-COVID

NAC has long been established as a mucolytic, reducing mucus viscosity through disulfide bond cleavage, yet its potential in chronic airway disease has been revisited through contemporary randomized trials. In mild-to-moderate COPD, a multicentre, double-blind, placebo-controlled trial (*n* = 968) testing high-dose NAC (600 mg twice daily) over two years did not reduce the overall exacerbation rate or improve FEV_1_, but subgroup analyses showed fewer moderate-to-severe exacerbations, particularly in GOLD stage 2 patients and those with lower symptom burden (mMRC < 2, CAT < 10) and/or prior exacerbations, suggesting a disease-stage-dependent effect.^61^ Complementary evidence comes from the NEWEST trial, in which nebulized NAC significantly improved phlegm severity scores over 12 weeks in patients with COPD,^62^ consistent with a local mucolytic effect and possibly enhanced drug delivery to the airway lumen. While inhaled NAC provides high local concentrations in airway mucus, NACA's systemic use may better address parenchymal oxidative injury where cell penetration limits NAC efficacy.

At the biomarker level, recent work shows that oral NAC reduces proinflammatory mediators such as IL-8 and eosinophil cationic protein while modulating antioxidant enzyme pathways, supporting a role in redox and inflammatory regulation.^63^ Post-viral syndromes, particularly long-COVID, are a natural extension of its redox-modulating profile. Persistent mitochondrial dysfunction, redox imbalance, and immune exhaustion have been described in post-viral states,^64^ and NAC may help restore thiol pools, rebalance mitochondrial bioenergetics, and support immune resilience.^65^*In vitro* studies show that NAC reduces inflammasome activation induced by SARS-CoV-2 proteins.^44^ Together, these findings suggest that NAC's utility in pulmonary disease is contingent on disease stage, symptom burden, and delivery route, opening opportunities for biomarker-guided stratification, with inflammatory mediators such as IL-8, eosinophil cationic protein, and exhaled 8-isoprostane emerging as candidate markers to identify responders.^63^

### 5.2 Hepatic medicine: acetaminophen overdose and NAFLD/NASH

The use of intravenous NAC as an antidote for acetaminophen overdose remains one of the best-established life-saving therapies in hepatology, where timely administration restores GSH and prevents acute liver failure. Beyond this classic indication, NAC has recently been tested in chronic liver disease. A six-month randomized controlled trial comparing NAC, rosuvastatin, and vitamin E in 135 patients with nonalcoholic steatohepatitis (NASH) demonstrated that NAC (1200 mg twice daily) reduced steatosis, lipid peroxidation, and inflammatory cytokines while modulating biomarkers such as FGF21, TIMP-1, and PIIINP, which are associated with fibrosis and metabolic stress.^66^ These findings support exploring a biomarker-guided approach, as NAC appears to act on specific disease pathways that vary in prominence across patients. Pharmacogenetic variation in glutathione-pathway genes is relevant to liver disease biology. The GCLC −129C/T promoter polymorphism has been independently associated with biopsy-proven non-alcoholic steatohepatitis (NASH).^67^ In acetaminophen poisoning, GSTT1/GSTM1/GSTP1 variants correlate with severity markers.^68^ However, genotype-stratified NAC treatment trials remain scarce. The integration of omics signatures, transcriptomic and metabolomic profiles of redox imbalance, may represent a promising, though still exploratory, route to stratifying NAC therapy in hepatology, where biomarkers such as FGF21, TIMP-1, PIIINP, and GSH pathway genotypes may help define responsive subgroups.^66^ For chronic liver disease where long-term dosing is contemplated, a NACA strategy achieving target engagement at lower oral doses could improve adherence and GI tolerability relative to high-dose NAC.

### 5.3 Neurology and psychiatry: addiction, schizophrenia, bipolar disorder, neuroinflammation, and neurodegeneration

In neuropsychiatry, NAC has been investigated as an adjunctive agent targeting glutamatergic dysregulation and oxidative stress. In cocaine dependence, MRS studies have shown that NAC normalizes elevated anterior cingulate glutamate, directly linking cysteine supplementation to central neurotransmitter modulation.^57^ In schizophrenia, adjunctive NAC increased medial prefrontal GSH as measured by MRS, with parallel effects on cortical glutamate/glutamine ratios.^39^ More recently, metabolomic analyses in bipolar disorder have suggested that baseline amino acid and oxidative stress signatures may predict response to NAC adjunctive therapy.^69^ These emerging findings point toward interindividual variability in neurotransmitter and redox networks as key determinants of efficacy. The incorporation of transcriptomic and metabolomic predictors into clinical trials could enable a transition from heterogeneous results to biomarker-stratified efficacy in neuropsychiatry. Brain MRS glutamate/GSH ratios, plasma amino acid profiles, and oxidative stress metabolomic signatures may serve as predictors of response in psychiatric populations.^69^

Neuroinflammation and neurodegeneration are another promising frontier, as redox-sensitive pathways such as HMGB1 *S*-nitrosylation have been implicated in neuroinflammation,^55^ though direct NAC evidence comes from other preclinical studies. In neurodegenerative models, for example in Parkinson's disease 6-OHDA rats, NAC preserves dopaminergic neuron viability, restores dopamine transporter expression, and ameliorates motor deficits.^70^ In models of cognitive aging and Alzheimer's disease, NAC has been shown to increase glutathione, reduce oxidative damage, and in some small trials improve cognitive function or slow decline.^71,72^ In ferroptosis-related disorders such as stroke and traumatic brain injury, NAC acts as a cysteine donor to sustain GSH peroxidase 4 (GPX4) activity, thereby reducing ferroptotic cell death.^49^ Because CNS exposure constrains many negative or equivocal NAC trials, NACA's brain penetration and BBB-stabilizing effects support prioritized evaluation in neurodegenerative and neuropsychiatric indications.^30,31^

Building on preclinical insights, our group demonstrated that NAC disrupts cystatin C amyloid complex formation *via* direct thiol interaction with amyloidogenic proteins *in vitro* and in patient-derived skin models of hereditary cystatin C amyloid angiopathy (HCCAA), establishing a mechanistic rationale for clinical testing.^73^ This was subsequently advanced into a nonrandomized clinical trial in HCCAA patients, where oral NAC reduced amyloid aggregation markers and showed signals of clinical benefit.^74^ Although exploratory in design, this represents one of the rare instances in which NAC has moved from mechanistic discovery to targeted clinical application. In parallel, our group tested the glutamatergic modulator fasoracetam in adolescents with ADHD carrying mGluR network variants, demonstrating a precision-medicine approach to glutamatergic modulation in psychiatry.^75^ Although fasoracetam is mechanistically distinct from NAC, the study highlights the principle that redox- and glutamate-targeting agents may yield benefit in genetically defined subgroups.

### 5.4 Endocrine and metabolic health: diabetes, obesity, insulin resistance

Preclinical studies consistently show that NAC ameliorates metabolic stress by restoring GSH, improving mitochondrial biogenesis *via* the PGC-1α/SIRT1 axis, and reducing lipid peroxidation and inflammation.^76^ Human data remain limited, but small trials in metabolic syndrome and obesity report improvements in oxidative stress markers and insulin sensitivity.^77,78^ The variability of NAC pharmacokinetics under conditions of altered metabolism, including obesity and diabetes, remains incompletely defined and represents an important precision-medicine gap.^20^ Circulating biomarkers of oxidative damage (malondialdehyde, 4-HNE), insulin resistance indices (*e.g.*, HOMA-IR), and cysteine/cystine redox balance may provide tools for identifying patients most likely to benefit, though large stratified trials are needed. Where intracellular redox is the proximal target (*e.g.*, myocyte/adipocyte mitochondria), NACA could achieve greater tissue-level thiol repletion at lower doses than NAC.

### 5.5 Cardiovascular disease: endothelial dysfunction, oxidative stress, atherosclerosis

Evidence for NAC in cardiovascular disease remains more limited than in hepatic or pulmonary settings, but several mechanistic studies support potential vascular benefits *via* redox modulation. Andrews *et al.* (2001) demonstrated that NAC acutely improves endothelium-dependent vasodilation, both coronary and peripheral, in patients with coronary artery disease, by enhancing acetylcholine-mediated vasomotor function, likely through reducing oxidative inactivation of nitric oxide.^79^ Lu *et al.* (2001) similarly showed that NAC attenuates the drop in microcirculatory flow caused by cigarette smoking in healthy volunteers, consistent with an antioxidant protective mechanism.^80^ Genetic variability may also play a role: GSH *S*-transferase (GST) polymorphisms, including GSTT1, GSTM1, and GSTP1, have been associated with increased cardiovascular risk and altered oxidative stress susceptibility.^81^ For example, Sombié *et al.* (2020) reported a significant association between the GSTT1-null genotype and essential hypertension in a Burkinabe cohort.^82^ Advances in redox proteomics now permit precise mapping of vascular thiol oxidation states and identification of oxidized cysteine residues, offering a potential platform for patient stratification and monitoring, though these approaches remain largely investigational in the cardiovascular domain. GST polymorphisms (GSTM1, GSTT1, GSTP1), vascular redox proteomic signatures, and nitric oxide bioavailability metrics may be used for precision cardiovascular trials with NAC.^81,82^ Given endothelium-focused mechanisms, improved endothelial cell uptake with NACA may augment vasoprotective redox effects observed with NAC.

### 5.6 Oncology: chemoprotection *versus* tumor-supportive effects

In oncology, NAC occupies a paradoxical role. On one hand, it protects normal tissues from chemotherapy-induced oxidative stress and has been shown in preclinical models to prevent ferroptotic death in healthy cells by replenishing cysteine and supporting GPX4 activity.^49^ The tumor biology paradox remains a critical limitation: while NAC protects normal tissues from oxidative stress and ferroptosis, it may also strengthen the redox defenses of tumors with high SLC7A11 or NRF2 activity, thereby promoting therapy resistance.^45^ On the other hand, tumors with high activity of the SLC7A11 cystine transporter or NRF2 pathway may exploit NAC supplementation to enhance redox defense, potentially blunting therapy efficacy. Recent work in triple-negative breast cancer, for instance, demonstrates that cysteine availability, sustained through uptake, drives NRF2 activation and oxidative stress resistance.^45^ Cancer immunotherapy also represents an untapped opportunity: by modulating T-cell activation thresholds, cytokine signaling, and oxidative stress in the tumor microenvironment, NAC synergized with PD-1 blockade in preclinical colorectal cancer models,^46^ though this requires careful balancing to avoid blunting cytotoxic ROS. Precision oncology approaches are therefore warranted: stratifying patients by SLC7A11 and NRF2 status, integrating tumor redox biomarkers such as lipid peroxides and glutathione redox state, and embedding biomarker enrichment in trial design. Without such approaches, NAC's role in cancer will remain uncertain, oscillating between protective adjuvant and potential tumor shield. Any consideration of NACA in oncology should mirror the caution applied to NAC: superior cellular access may intensify both chemoprotective benefits in normal tissues and, in some tumors with high SLC7A11/NRF2 activity, unwanted reinforcement of redox defenses; biomarker gating will be essential.

### 5.7 Other emerging areas: pharmacological innovations, aging, microbiome, and toxicology

Beyond the clinical domains described above, several additional frontiers for NAC are emerging. Advances in formulation science, including liposomal and other carrier-based NAC preparations, prodrug derivatives such as NACA, and modified-release approaches, have been explored to improve cellular delivery and target engagement in specific experimental settings.^83^ In aging research, NAC attenuated sarcopenia in chronic liver disease-associated models, improving mitochondrial quality control and counteracting oxidative stress-driven muscle loss.^84^ Combination thiol strategies such as GlyNAC are particularly relevant to aging research, where mitochondrial decline and redox imbalance are prominent features.^23^ By targeting multiple nodes of the thiol–redox network simultaneously, these approaches may offer broader benefits than NAC alone.

Evidence from both gut and infection models shows that NAC can disrupt or prevent bacterial biofilms, influence microbiome composition, and modulate mucosal oxidative stress and immune signaling. For instance, NAC inhibited crystalline biofilm formation by *Proteus mirabilis* in catheter models,^85^ reduced inflammation in bladder epithelial cells, and in autoimmune mouse models reshaped gut microbiota while lowering mucosal oxidative stress and systemic autoimmunity.^86^ Environmental and occupational toxicology also warrant attention, as NAC has demonstrated effects on copper and zinc homeostasis^87^ and has been reported to mitigate damage from air pollution and heavy metals.^88,89^ These lines of investigation extend NAC research beyond its established indications into models of host defense, environmental exposure, and microbiome-associated inflammation, although clinical validation in these areas remains limited.^12^ Formulation advances that benefitted NAC (*e.g.*, liposomes, nanocarriers) are likely synergistic with NACA's chemistry, potentially enabling ultra-low dosing with targeted delivery.

## 6. Challenges and critical perspectives

Despite broad opportunities, significant challenges constrain the clinical translation of NAC. Excessive reduction, or reductive stress, is a conceptual risk, particularly when high exposures are achieved without biomarker confirmation of oxidative stress or target engagement.^22^ Clinical outcomes remain variable across trials, reflecting heterogeneity in patient populations, baseline oxidative states, and delivery routes.^12^ Pharmacological constraints persist, with low oral bioavailability and route-dependent systemic exposure limiting consistent tissue delivery.^20^ A frequently underappreciated source of heterogeneity is renal function. Although only a small fraction of NAC is excreted unchanged in urine, kidney function influences NAC pharmacokinetics, and available data indicate that total NAC clearance is reduced in advanced CKD, with consequent changes in systemic exposure relative to individuals with preserved renal function.^22^ Reduced renal function is also accompanied by characteristic disturbances in thiol–redox biology, including glutathione deficiency and altered Nrf2-dependent signaling, which may shift the balance of NAC's molecular effects and the margin between target engagement and reductive stress.^22^ Given the high prevalence of CKD comorbidity across many indications in which NAC has been proposed or tested, renal function should be treated as a key design and stratification variable in future PK/PD-informed trials.

NACA was designed to address several pharmacological bottlenecks of NAC, *e.g.*, oral bioavailability, tissue penetration, and CNS access, yet its clinical dose–exposure–response relationships remain to be defined. Safety concerns, though generally modest, include gastrointestinal side effects, anaphylactoid reactions with intravenous use, and uncertainties surrounding chronic high-dose exposure.^26^ Regulatory ambiguity further complicates translation, as NAC straddles a dual identity as both drug and supplement, raising issues of standardization, quality control, and consistent dosing guidelines across jurisdictions.^1^

Further progress depends on matching NAC's thiol pharmacology to measurable target engagement and disease-relevant endpoints, rather than treating it as a generic antioxidant. Beyond the traditional antioxidant framing, NAC should be viewed as a systems-level rheostat of thiol–redox homeostasis, capable of tuning the cysteine–GSH–thiol proteome axis in a context-dependent manner. This perspective implies that responses are not binary but network-determined, shaped by baseline proteomic wiring, mitochondrial function, and ferroptotic sensitivity. In parallel, NAC may be considered as a metabolic checkpoint modulator of immunity, recalibrating T-cell activation thresholds, cytokine signaling, and redox balance in ways that could synergize with immunotherapies or restore post-viral immune competence.

Realizing these visions will require addressing bottlenecks: nonspecificity of action, inconsistent trial outcomes, lack of biomarker-guided stratification, and limited pharmacological optimization. Approaches that improve target engagement and strengthen interpretability of clinical signals are likely to be most informative for future trial design. On the pharmacological side, formulation and delivery strategies such as liposomal and other carrier-based NAC preparations may enhance intracellular delivery and cellular target engagement in relevant contexts.^83^ Mechanistically, redox-proteomic platforms are increasingly able to map context-dependent cysteine oxidation states and reversible thiol modifications, providing tools to quantify redox target engagement and downstream pathway modulation.^53^ On the inference side, causal frameworks including Mendelian randomization can complement conventional trials by testing whether genetically proxied variation in redox-related pathways is consistent with disease modification, thereby helping prioritize indications and endpoints for interventional studies.^90^ Most importantly, future trials must move beyond broad inclusion and instead embed precise phenotyping and patient selection strategies. Incorporating pharmacogenomics, biomarker stratification, and mechanistic endpoints into trial design will allow NAC to be used not as a blunt intervention but as a probe of thiol–redox circuitry and cysteine redox control across biological networks.^25,52^ For instance, future trials could stratify cardiovascular patients by GST polymorphisms, oncology cohorts by SLC7A11/GPX4 expression, or psychiatric patients by metabolomic redox signatures. Such biomarker-enriched frameworks would define responder groups, refine dosing strategies, and avoid unintended harms, positioning NAC as a model candidate for next-generation precision thiol therapeutics. Early-phase NACA studies should pre-specify PK/PD targets (intracellular GSH, lipid peroxidation markers), incorporate CNS-accessible readouts where relevant (MRS GSH/Glx), and apply the same biomarker-guided stratification proposed for NAC.

## Author contributions

Hui-Qi Qu: conceptualization; literature review; figure preparation; writing – original draft. Charlly Kao: writing – review & editing. Hakon Hakonarson: conceptualization; supervision; writing – review & editing; critical revision. All authors have read and approved the final version of the manuscript.

## Conflicts of interest

Drs. Kao and Hakonarson reported pending patents for *N*-acetylcysteine and its derivatives, as therapy for hereditary cystatin C amyloid angiopathy and Alzheimer's disease, and Dr. Hakonarson and Dr. Kao own equity in Arctic Therapeutics, developing NAC and NACA for dementia. No other disclosures were reported. The authors declared no other conflicts of interest with respect to the research, authorship, and/or publication of this article.

## Data Availability

No primary research results, software or code have been included and no new data were generated or analysed as part of this review.
